# Supplementary material for: The genome sequence of the Golden-tabbed robberfly, Eutolmus rufibarbis (Meigen, 1820)
Source: Wellcome Open Res. 2024 Oct 15;9:593. [Version 1] doi: 10.12688/wellcomeopenres.23162.1 (PMC11602699; doi:10.12688/wellcomeopenres.23162.1)
Supplement: Peer review history

# Reviewer report: Reviewer response for version 1

**DOI:** 10.21956/wellcomeopenres.25506.r109719

**Published:** 2024-11-27

**Authors:** Daniel Bruzzese

**Affiliations:** 1 Yale University, New Haven, Connecticut, USA

The authors generated an exceptional genome assembly for the Golden-tabbed robberfly. The final assembly had a total length of ~286 megabases and most of it scaffolded into 7 fragments. They also produced the mitochondrial genome. They were thorough in demonstrating how well the near-chromosomally complete genome was assembled and provided detailed methods for its construction. Very nice!

Are sufficient details of methods and materials provided to allow replication by others?

Yes

Is the rationale for creating the dataset(s) clearly described?

Yes

Are the datasets clearly presented in a useable and accessible format?

Yes

Are the protocols appropriate and is the work technically sound?

Yes

Reviewer Expertise:

Evolutionary biology, symbiosis, vector biology, bioinformatics, and genomics.

I confirm that I have read this submission and believe that I have an appropriate level of expertise to confirm that it is of an acceptable scientific standard.

# Reviewer report: Reviewer response for version 1

**DOI:** 10.21956/wellcomeopenres.25506.r108233

**Published:** 2024-11-02

**Authors:** Jaakko Pohjoismäki

**Affiliations:** 1 University of Eastern Finland, Joensuu, Finland

The data note by Will Nash, Andrew Halstead and the DToL consortium presents a reference genome assembly for a species of robberflies, *Eutolmus rufibarbis*. The assembly meets the EBP standards and represents a really nice addition to the growing list of Dipteran reference genomes, where the so-called "lower brachycera" have been somewhat underrepresented.

 The manuscript is technically sound and detailed, and the described assembly already with relevant metadata are accessible through the given reference numbers. The presented genome assembly is highly continuous and complete. Although the specimen images are a bit suboptimal, I have no doubt of the identification, which has been also checked by DNA barcoding.

 I have only few minor suggestions:

**Background**

 1) What is meant by robber flies sitting "at the top level of the UK’s entomological safari"? One can guess, but this could be elaborated for the general interests of the readers.

 2) *Didysmachus picipes* has also uniformly black legs and leg bristles, and is superficially quite similar. The species can be differentiated based on the relative length of their dc bristles (among other things), as *picipes* belongs to the *Dysmachus* and *Eutolmus* to the *Machimus*-group.

**Genome sequence report**

 I note that the genome assembly can be already accessed through NCBIs genome viewer. I was delighted to see that even the gene names (where available) were visible in the annotation, and not just loci numbers, as in many cases for such insects. However, I also noted that the mtDNA annotations are missing? This can be a NCBI issue, but please check. 

**Methods**

 1) Too bad that only a female specimen was sequenced and not the heterogametic male sex.

 It was a pleasure to see an asilid genome, thank you for the release.

Are sufficient details of methods and materials provided to allow replication by others?

Yes

Is the rationale for creating the dataset(s) clearly described?

Yes

Are the datasets clearly presented in a useable and accessible format?

Yes

Are the protocols appropriate and is the work technically sound?

Yes

Reviewer Expertise:

molecular biology, biodiversity genetics, taxonomy

I confirm that I have read this submission and believe that I have an appropriate level of expertise to confirm that it is of an acceptable scientific standard.
